# Moderate‐intensity training in hypoxia improves exercise performance and glycolytic capacity of skeletal muscle in horses

**DOI:** 10.14814/phy2.15145

**Published:** 2021-12-10

**Authors:** Kazutaka Mukai, Yu Kitaoka, Yuji Takahashi, Toshiyuki Takahashi, Kenya Takahashi, Hajime Ohmura

**Affiliations:** ^1^ Sports Science Division Equine Research Institute Japan Racing Association Shimotsuke Tochigi Japan; ^2^ Department of Human Sciences Kanagawa University Yokohama Kanagawa Japan; ^3^ Department of Sports Sciences University of Tokyo Tokyo Japan

**Keywords:** glycolytic capacity, horse, hypoxic training, performance

## Abstract

We investigated whether moderate‐intensity training of horses in moderate hypoxia for 4 weeks elicits greater adaptations in exercise performance, aerobic capacity, and glycolytic/oxidative metabolism in skeletal muscle compared to normoxic training. In a randomized crossover study design, seven untrained Thoroughbred horses (5.9 ± 1.1 years, 508 ± 9 kg) completed 4 weeks (3 sessions/week) of two training protocols consisting of 3‐min cantering at 70% of maximal oxygen consumption ($\dot{V}O_{2\max}$) in hypoxia (HYP; *F*_I_O_2_ = 14.7%) and normoxia (NOR; *F*_I_O_2_ = 21.0%) with a 4‐month washout period. Normoxic incremental exercise tests (IET) were conducted before and after training. Biopsy samples were obtained from the middle gluteal muscle before IET and monocarboxylate transporter (MCT) protein expression and glycolytic/mitochondrial enzyme activities were analyzed. Data were analyzed using mixed models (*p* < 0.05). Running speed was 7.9 ± 0.2 m/s in both groups and arterial oxygen saturation during training in NOR and HYP were 92.9 ± 0.9% and 75.7 ± 3.9%, respectively. Run time in HYP (+9.7%) and $\dot{V}O_{2\max}$ in both groups (NOR, +6.4%; HYP, +4.3%) at IET increased after 4 weeks of training. However, cardiac output, arterial‐mixed venous O_2_ difference, and hemoglobin concentration at exhaustion were unchanged in both conditions. While MCT1 protein and citrate synthase activity did not increase in both conditions after training, MCT4 protein (+13%), and phosphofructokinase activity (+42%) increased only in HYP. In conclusion, 4 weeks of moderate‐intensity hypoxic training improves exercise performance and glycolytic capacity of skeletal muscle in horses.

## Graphical abstract

Four weeks of moderate‐intensity training in hypoxia (70% $\dot{V}O_{2\max}$ 3 min, 3 sessions/week, *F*_I_O_2_ = 14.7%) increased run time and $\dot{V}O_{2\max}$ at incremental exercise tests, monocarboxylate transporter (MCT) 4 protein content and phosphofructokinase (PFK) activity of skeletal muscle in horses, whereas the same training in normoxia only increased $\dot{V}O_{2\max}$.

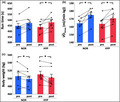

## 1 INTRODUCTION

Hypoxic training has gained popularity in athletes, especially in endurance athletes, to improve their endurance exercise performance and aerobic capacity. There are three major hypoxic training programs: live high‐train high (LHTH), live high‐train low (LHTL), and live low‐train high (LLTH) (Wilber, 2007). LHTH and LHTL programs focus mainly on improving endurance performance, aerobic capacity, and erythrocyte volume/hemoglobin mass (Sinex & Chapman, 2015; Wilber, 2007), whereas LLTH program aims to enhance not only endurance performance and aerobic capacity, but also sprint performance and glycolytic capacity (Millet & Girard, 2017; Wilber, 2007). In LLTH training, exercise in hypoxia reduces O_2_ delivery to skeletal muscle and increases glycolytic energy contribution. These hypoxic conditions in muscle tissue may stimulate glycolytic pathways and induce greater adaptations in skeletal muscle compared to normoxic training (Faiss et al., 2013).

The oxygen consumption ($\dot{V}O_{2\max}$) and blood lactate concentration of Thoroughbred horses during intense exercise have often been reported as greater than 140 ml/(min kg) and 20 mmol/l, respectively (Kitaoka et al., 2012; Mukai et al., 2007, 2008). These data indicate that Thoroughbred horses utilize aerobic and glycolytic energy resources fully during maximal exercise, and the training strategy for racehorses is to improve both aerobic and glycolytic capacity. During maximal exercise, lactate is produced mainly in fast glycolytic muscle, is transported to slow oxidative muscle, which contains abundant mitochondria, and is utilized as a substrate (Brooks, 2009; Juel & Halestrap, 1999). Lactate transverses the plasma membrane of skeletal muscle fiber via proton‐linked monocarboxylate transporter (MCT) 1 and 4 (Bonen, 2001). MCT1 protein content increases after endurance training and correlates positively with citrate synthase (CS) activity, whereas MCT4 protein content does not increase after endurance training and shows no relationship with CS activity in humans (Dubouchaud et al., 2000). In horses, we have reported previously that MCT1 and MCT4 protein expression increased after 18 weeks of high‐intensity training and MCT1 protein expression was maintained following an additional 6 weeks of moderate‐intensity training, whereas MCT4 protein returned to the baseline level (Kitaoka et al., 2011). Furthermore, the MCT4 protein level was correlated positively with improvement of exercise performance after 6 weeks of high‐intensity training (Kitaoka et al., 2009), suggesting that MCT4 may play a pivotal role during high‐intensity exercise in horses.

Our previous studies demonstrate that high‐intensity training in hypoxia enhances exercise performance and aerobic capacity in horses compared with normoxic training (Mukai et al., 2020; Ohmura et al., 2017) and that 2 weeks of high‐intensity training in hypoxia increases MCT4 protein expression and the activity of phosphofructokinase (PFK), a major glycolytic enzyme (Wang et al., 2020). Collectively, hypoxic training may have a benefit to enhance both aerobic and glycolytic capacity, which is essential for racehorse training. However, we have observed greater weight loss after hypoxic training than after normoxic training (Mukai et al., 2021), and, thus, there is a need for lower intensity, but still effective hypoxic training for horses.

Therefore, the purpose of this study was to test whether horses trained at moderate intensity in moderate hypoxia for 4 weeks experience greater improvements in performance, aerobic capacity, and glycolytic/oxidative capacity in skeletal muscle compared to horses trained in normoxia.

## 2 MATERIALS AND METHODS

Protocols for the study were reviewed and approved by the Animal Welfare and Ethics Committee of the Japan Racing Association (JRA) Equine Research Institute (Permit number: 2019‐3 & 2019‐4). All incisions for catheter placements and muscle biopsies were performed under local anesthesia using lidocaine. All efforts were made to minimize animal suffering.

### 2.1 Horses

Seven untrained Thoroughbreds (5 geldings, 2 females; mean ± SE age, 5.9 ± 1.1 years; body weight, 508 ± 9 kg at the onset of the study) were used in this study. The horses had a carotid artery surgically moved from the carotid sheath to a subcutaneous location under sevoflurane anesthesia to facilitate arterial catheterization. After recovery from surgery, the horses were accustomed to running on a treadmill (Sato I, Sato AB, Uppsala, Sweden) while wearing an open‐flow mask (Pascoe et al., 1999). After the surgery, each horse was kept in a 17 × 22 m yard for approximately 6 h/day every day for at least 4 months before treadmill experiments began. All horses received 1 kg of oats, 1 kg of pelleted feed, and 3 kg of timothy hay in the morning and 1 kg of oats, 2 kg of pelleted feed, and 3 kg of timothy hay in the afternoon. Water was available ad libitum during the training period.

### 2.2 Experimental design

**FIGURE 1:**
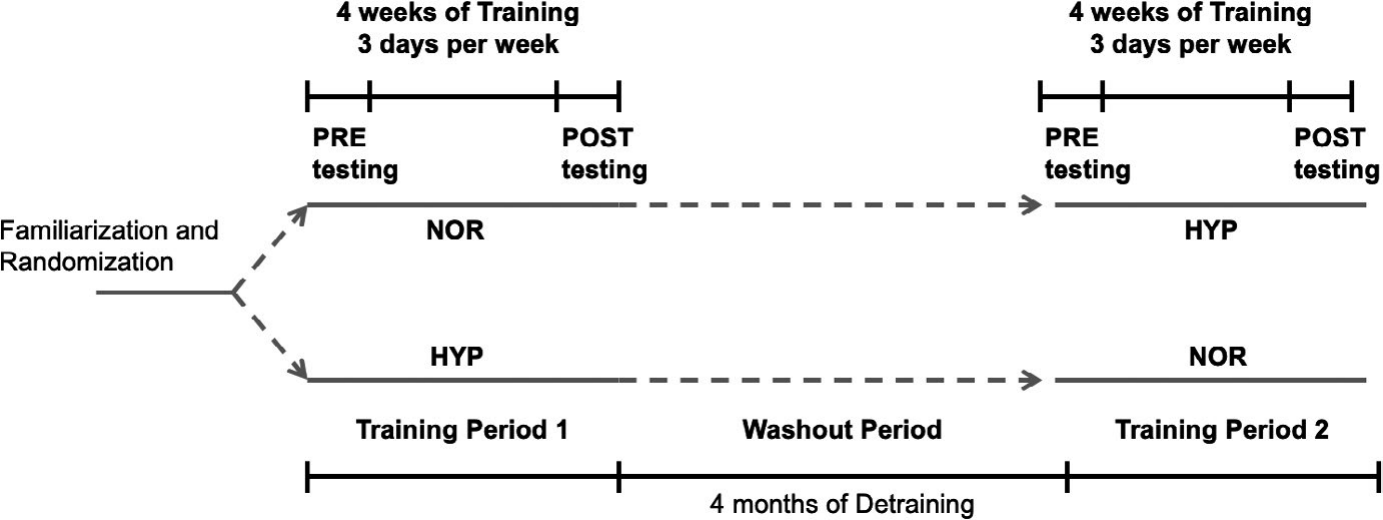
Experimental study design. In a randomized, crossover design, horses were trained in normoxia (NOR; *F*_I_O_2_ = 21.0%) or hypoxia (HYP; *F*_I_O_2_ = 14.7%) for 3 days/week for 4 weeks. Each training period was separated by 4 months to ensure a sufficient detraining interval

In a randomized crossover design, horses were trained in moderate hypoxia (HYP, *F*_I_O_2_ = 14.7%) and normoxia (NOR, *F*_I_O_2_ = 21.0%) at 70% $\dot{V}O_{2\max}$ for 3 min, 3 days/week for 4 weeks (Figure 1) and were pastured in the yards for approximately 6 h/day and walked for 1 h/day in a walker on the other 4 days during the training period. Each training period was separated by 4 months to ensure a sufficient detraining interval (Figure 1). The training session consisted of walking at 1.7 m/s for 30 min in a walker, trotting at 4 m/s for 2 min, and cantering at the speed to elicit 70% $\dot{V}O_{2\max}$ measured in normoxia for 3 min on a treadmill at a 6% incline, followed by 1.7 m/s for 30 min in a walker in both groups. In HYP, horses wore an open‐flow mask before trotting and were exposed to hypoxia during trotting for 2 min and cantering at 70% $\dot{V}O_{2\max}$ for 3 min. In NOR, horses did not wear a mask during training sessions.

### 2.3 Incremental exercise tests (IET) in normoxia

Incremental exercise tests in normoxia were conducted before and after training. We set the timing of the last exercise bout at 48 h before the post‐training IET and muscle biopsy to minimize an acute effect of exercise on skeletal muscle and to avoid a decrease in the training effects as demonstrated in previous training studies (Perry et al., 2010). The procedure for the incremental exercise test, including oxygen consumption measurements and blood sampling, has been described previously (Mukai et al., 2017). Briefly, after catheters and transducers were connected and tested, the horse began its exercise. The horse warmed up by trotting at 4 m/s for 3 min, then, an open‐flow mask was fitted to the horse and the horse began exercising up a 6% incline for 2 min each at 1.7, 4, 6, 8, 10, 12, 13, and 14 m/s until the horse could not maintain its position at the front of the treadmill with humane encouragement. This was defined as exhaustion. Run time to exhaustion was measured with a stopwatch. For each speed, $\dot{V}O_{2\max}$ was calculated for the final 30 s of each step. Heart rate was recorded using a commercial heart rate monitor (S810, Polar, Kempele, Finland), and mean heart rate was calculated for the final 30 s of each step.

### 2.4 Oxygen consumption at IET

Horses wore an open‐flow mask on the treadmill through which a rheostat‐controlled blower drew air. Air flows through a 25‐cm diameter tubing and across a pneumotachograph (LF‐150B, Vise Medical, Chiba, Japan) connected to a differential pressure transducer (TF‐5, Vise Medical, Chiba, Japan) to ensure that bias flows during measurements were identical to those used during calibrations. Bias flow was set to maintain changes in O_2_ concentration and CO_2_ concentrations at <1.5% to avoid having the horses rebreathe CO_2_. Oxygen and CO_2_ concentrations were measured using an O_2_ and CO_2_ analyzer (MG‐360, Vise Medical, Chiba, Japan), and calibrations were used to calculate rates of O_2_ consumption and CO_2_ production with mass flow meters (CR‐300, Kofloc, Kyoto, Japan) using the N_2_‐dilution/CO_2_‐addition mass‐balance technique (Fedak et al., 1981). Gas analyzer and mass flow meter outputs were also recorded on personal computers using commercial hardware and software (DI‐720 and Windaq Pro+, DATAQ, Akron, OH) with sampling at 200 Hz.

### 2.5 Blood sampling at IET

Before leading a horse onto the treadmill, an 18‐gauge catheter (Surflow, Terumo, Tokyo, Japan) was placed in the horse's carotid artery, and an 8‐F introducer (MO95H‐8, Baxter International, Deerfield, IL) was placed in the jugular vein. A Swan‐Ganz catheter (SP5107U, Becton, Dickinson and Company, Franklin Lakes, NJ) was passed via the jugular vein so that its tip was positioned in the pulmonary artery, confirmed by measuring pressure at its tip with a pressure transducer (P23XL, Becton, Dickinson and Company, Franklin Lakes, NJ). Mixed‐venous blood samples were drawn from the tip of the Swan‐Ganz catheter and arterial samples were drawn from the 18‐gauge carotid catheter at timed intervals into heparinized syringes for the final 30 s of each step and at 1, 3, and 5 min after exhaustion, and were stored on ice until measured immediately following the experiment. Blood samples were analyzed with a blood gas analyzer (ABL800 FLEX, Radiometer, Copenhagen, Denmark) and, for O_2_ saturation (*S*O_2_) and concentration (*C*O_2_), with a hemoximeter (ABL80 FLEX‐CO‐OX, Radiometer, Copenhagen, Denmark). Following measurement of blood gases and oximetry, the blood was sampled for plasma lactate concentration with a lactate analyzer (Biosen S‐Line, EKF‐diagnostic GmbH, Barleben, Germany) after being centrifuged at 1740 × g for 10 min. The speed at which the plasma lactate concentration reached 4 mmol/l (*V*_LA4_) was calculated using exponential curve fitting. The Swan‐Ganz catheter in the pulmonary artery was connected to a cardiac output computer (COM‐2, Baxter International, Deerfield, IL) so that its thermistor registered pulmonary arterial temperature, which was recorded at each blood sampling and used to correct the blood gas measurements.

### 2.6 Hypoxic training protocol and measurements during exercise in the first week of each training period

The procedure for producing the hypoxic condition was slightly modified from the method previously described (Ohmura et al., 2010). Briefly, a mixing chamber was connected to the upstream flexible tube on a 25‐cm diameter open‐flow mask through which a flow of N_2_ was blown into the upstream end of the flow system and mixed with a bias‐flow of air of 80–120 l/s to create the desired inspired O_2_ concentration. Nitrogen gas flow was controlled with a mass flow meter (Model DPM3, Kofloc, Kyoto, Japan) connected to compressed gas cylinders through a gas manifold. Nitrogen gas flow was adjusted to maintain 15% O_2_ by monitoring the O_2_ concentration in the downstream arm of the mass flow meter with an O_2_ analyzer (LC‐240UW, Vise Medical, Chiba, Japan) when horses ran in hypoxia.

In the first week of training for all groups, we collected arterial blood samples in the final 15‐s of cantering at 70% $\dot{V}O_{2\max}$ during the exercise session to measure arterial blood gas variables (ABL800 FLEX and ABL80 FLEX‐CO‐OX, Radiometer, Copenhagen, Denmark) and plasma lactate concentration (Biosen S‐Line, EKF‐diagnostic GmbH, Barleben, Germany). We also recorded heart rate (S810, Polar, Kempele, Finland) during cantering.

### 2.7 Muscle sampling

Muscle samples were obtained from the middle gluteal muscle on an imaginary line drawn from the coxal tuber to the root of the tail, at one‐third the distance from the coxal tuber and at the same depth (5 cm from the skin surface) by needle biopsy under local anesthesia (lidocaine, Fujisawa Pharmaceutical Co., Osaka, Japan) immediately before incremental exercise tests. All muscle samples were frozen by liquid nitrogen and stored at −80°C until analyzed.

### 2.8 Western blotting

The procedure for western blotting was described previously (Wang et al., 2020). Briefly, the gluteus medius muscle sample was homogenized in radioimmunoprecipitation assay buffer (25 mmol/l Tris‐HCl, pH 7.6, 150 mmol/l NaCl, 1% NP‐40, 1% sodium deoxycholate, and 0.1% sodium dodecyl sulfate [SDS]) supplemented with protease inhibitor mixture (Complete Mini, ETDA‐free, Roche Applied Science, Indianapolis, IN) and phosphatase inhibitor mixture (PhosSTOP, Roche Applied Science). The total protein content of samples was quantified using the BCA protein assay (Pierce Biotechnology, Rockford, IL). Equal amounts of protein were loaded onto 12% SDS‐PAGE gels and separated by electrophoresis. Proteins were transferred to polyvinylidene difluoride membranes using the Trans‐Blot Turbo Transfer System (Bio‐Rad). The membranes were blocked in PVDF blocking reagent (TOYOBO, Osaka, Japan) for 1 h at room temperature (RT), followed by overnight incubation at 4°C with primary antibodies raised in rabbits against the oligopeptide corresponding to the C‐terminal regions of equine MCT1 and MCT4 (Cosmo Bio, Tokyo, Japan). Following washing in Tris‐Buffered Saline with Tween 20 (TBST), secondary antibody incubation was performed using the appropriate anti‐rabbit antibody for 1 h at RT. The membranes were washed again in TBST, then developed with chemiluminescent reagents. Bands were detected using the C‐DiGit Blot Scanner (LI‐COR, Lincoln, NE). Ponceau staining was used to verify consistent loading.

### 2.9 Enzyme activity

The gluteus medius muscle sample was homogenized in 100 ml (v/w) of 100 mmol/l potassium phosphate buffer. Activities of PFK, CS, and cytochrome *c* oxidase (COX) were measured spectrophotometrically to determine the glycolytic and oxidative capacities, respectively, following established protocols (Shonk & Boxer, 1964; Spinazzi et al., 2012).

### 2.10 Statistical analysis

Data are presented as mean ± standard error (SE). Differences in the variables between NOR and HYP during training sessions in the first week were analyzed using paired *t*‐tests. After training, the differences (time × group) were analyzed using mixed models. Tukey's tests were used as post hoc tests. Statistical analyses were performed with commercial software (JMP 13.1.0, SAS Institute Inc., Cary, NC) with significance defined as *p* < 0.05 .

## 3 RESULTS

### 3.1 Blood gas variables, heart rate, and plasma lactate concentration during exercise sessions in the first week of training

Mean O_2_ concentration during training sessions in HYP was 14.7 ± 0.1% and running speed was 7.9 ± 0.2 m/s in both groups. Blood gas variables, heart rate, and plasma lactate concentration at the end of exercise sessions are presented in Table 1. *S*_a_O_2_ and *P*_a_O_2_ in HYP were lower than those in NOR (*S*_a_O_2_, *p* = 0.0019, statistical power = 0.98; *P*_a_O_2_, *p* < 0.0001, statistical power = 1.00), but heart rate, plasma lactate concentration, and *P*_a_CO_2_ were not different between the groups (heart rate, *p* = 0.85, statistical power = 0.053; plasma lactate concentration, *p* = 0.16, statistical power = 0.19; *P*_a_CO_2_, *p* = 0.077, statistical power = 0.37) (Table 1).

**TABLE 1:** Parameters on aerobic capacity and blood gas analysis during exercise sessions at the 1st week of training

	NOR	HYP
*S*_a_O_2_ (%)	92.9 ± 0.9	75.7 ± 3.9^a^
*P*_a_O_2_ (Torr)	94.1 ± 3.6	47.6 ± 2.6^a^
*P*_a_CO_2_ (Torr)	45.1 ± 1.4	51.8 ± 3.6
Heart rate (bpm)	192 ± 6	193 ± 2
Plasma lactate concentration (mmol/l)	10.6 ± 2.5	7.0 ± 1.7

Note

Arterial O_2_ saturation (*S*_a_O_2_), arterial O_2_ partial pressure (*P*_a_O_2_), arterial carbon dioxide partial pressure (*P*_a_CO_2_), heart rate, and plasma lactate concentration at the end of exercise in the 1st week of training. Values are means ± SE for seven horses.

^a^ Significant differences from NOR (*p* < 0.05).

### 3.2 Effects of normoxic and hypoxic training on exercise performance and aerobic capacity at IET

**FIGURE 2:**
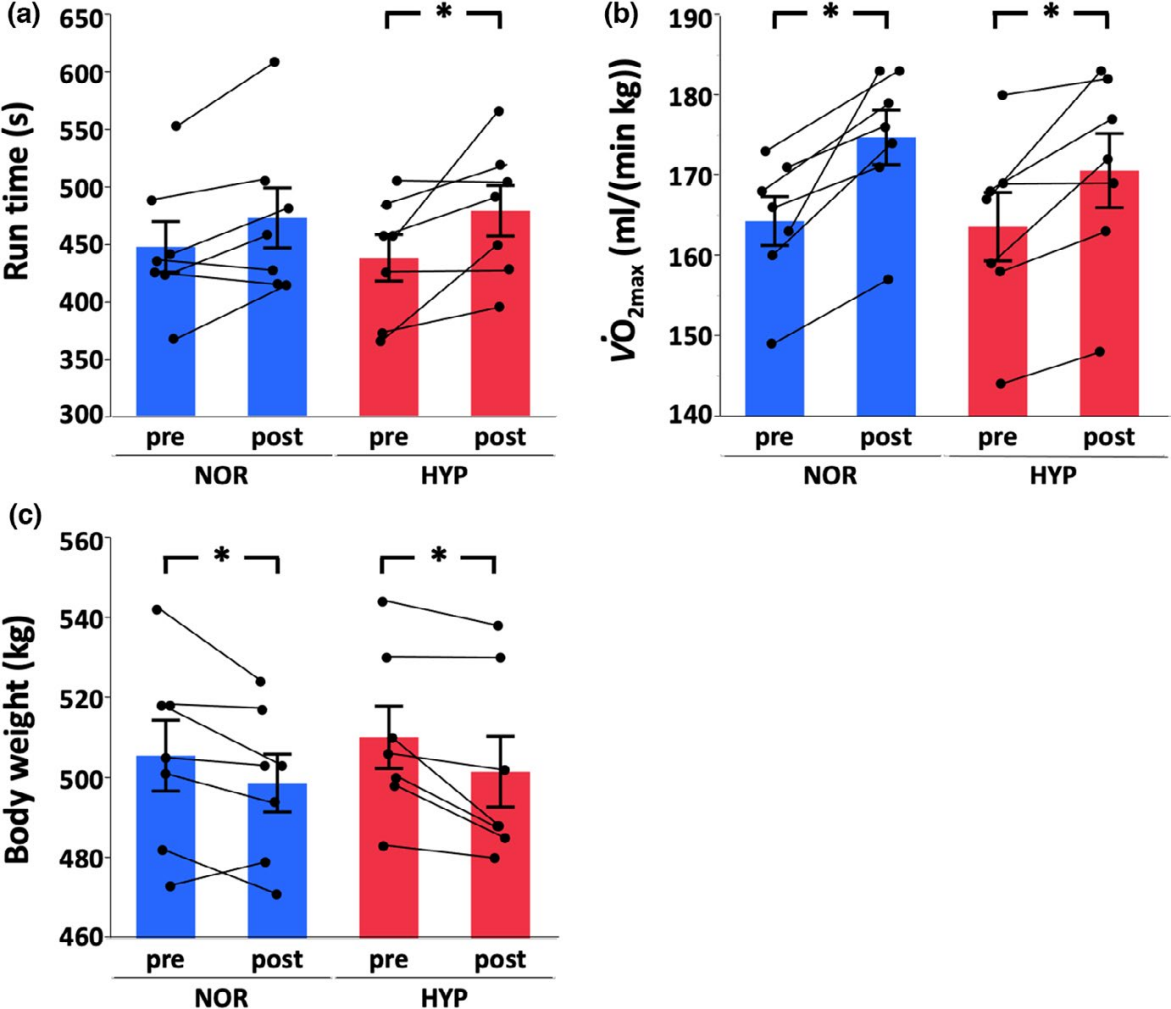
Run time (a), $\dot{V}O_{2\max}$_,_ (b) and body weight (c) in IET at pre‐training and post‐training in normoxia (NOR) or hypoxia (HYP). Values are mean ± SE. *Significant changes from pre‐training (*p* < 0.05)

**FIGURE 3:**
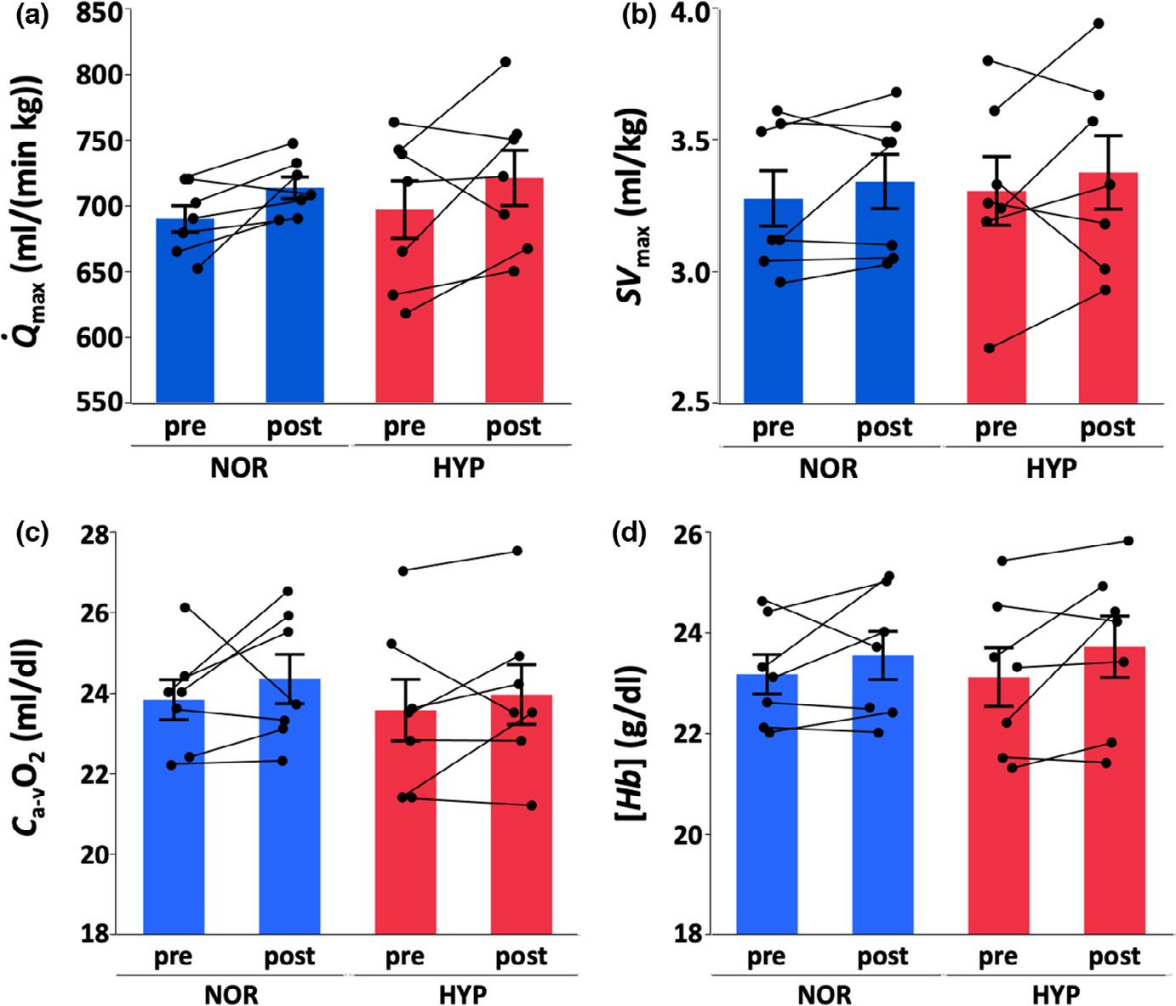
Cardiac output (${\dot{Q}}_{\text{max}}$; a), stroke volume (*SV*_max_; b), arterial‐mixed venous O_2_ difference (*C*_a‐v_O_2_; c), and hemoglobin concentration ([*Hb*], d) at exhaustion in IET at pre‐training and post‐training in normoxia (NOR) or hypoxia (HYP). Values are mean ± SE

**FIGURE 4:**
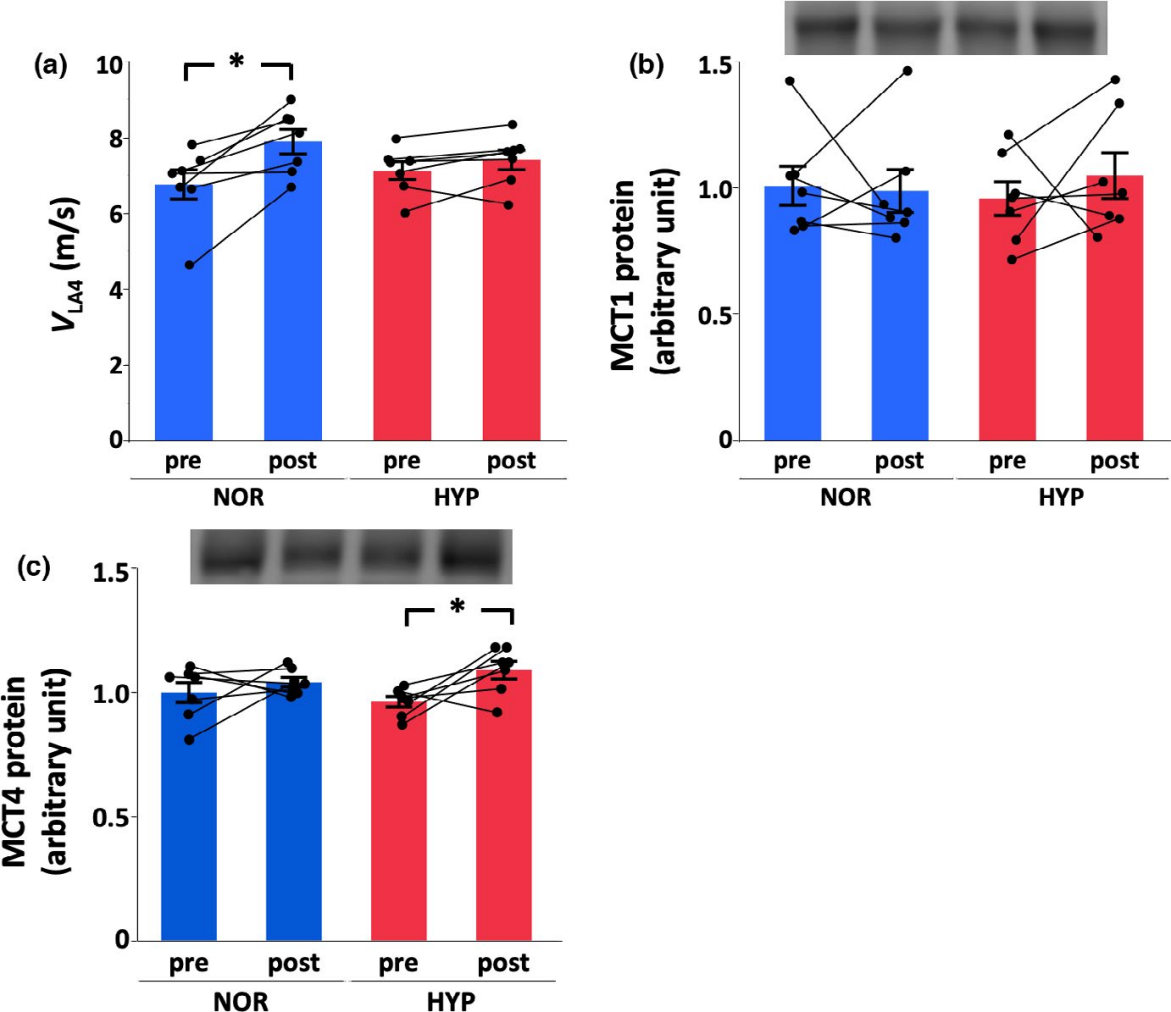
Speed at which plasma lactate concentration reached 4 mmol/l (*V*_LA4_, a), monocarboxylate transporter (MCT) 1 (b) and MCT4 (c) protein contents at pre‐training and post‐training in normoxia (NOR) or hypoxia (HYP). Values are mean ± SE. *Significant changes from pre‐training (*p* < 0.05)

After 4 weeks of training, run time increased only in HYP (NOR, +5.7%, *p* = 0.082, statistical power = 0.51; HYP, +9.7%, *p* = 0.0095, statistical power = 0.60), $\dot{V}O_{2\max}$ increased in both groups (NOR, +6.4%, *p* = 0.0004, statistical power = 0.99; HYP, +4.3%, *p* = 0.0063, statistical power = 0.75) and body weight decreased in both groups (NOR, −1.3%, *p* = 0.043, statistical power = 0.44; HYP, −1.7%, *p* = 0.015, statistical power = 0.71) (Figure 2). However, cardiac output (*Q*_max_, NOR, +3.5%, *p* = 0.13, statistical power = 0.54; HYP, +3.7%, *p* = 0.12, statistical power = 0.21), stroke volume (*SV*_max_, NOR, +2.1%, *p* = 0.43, statistical power = 0.15; HYP, +3.2%, *p* = 0.39, statistical power = 0.096), arterial‐mixed venous O_2_ difference (*C*_a‐v_O_2_, NOR, +2.3%, *p* = 0.36, statistical power = 0.11; HYP, +1.7%, *p* = 0.49, statistical power = 0.11) and hemoglobin concentration ([*Hb*], NOR, +1.6%, *p* = 0.28, statistical power = 0.16; HYP, +2.6%, *p* = 0.10, statistical power = 0.32) at exhaustion did not significantly change after training in both groups (Figure 3). In contrast, *V*_LA4_ increased only in NOR (NOR, +18.3%, *p* = 0.0006, statistical power = 0.87; HYP, +4.1%, *p* = 0.27, statistical power = 0.30) (Figure 4). However, there were no significant differences in any variables between groups.

### 3.3 Effects of hypoxic training on MCT protein expressions and glycolytic and mitochondrial enzyme activities

**FIGURE 5:**
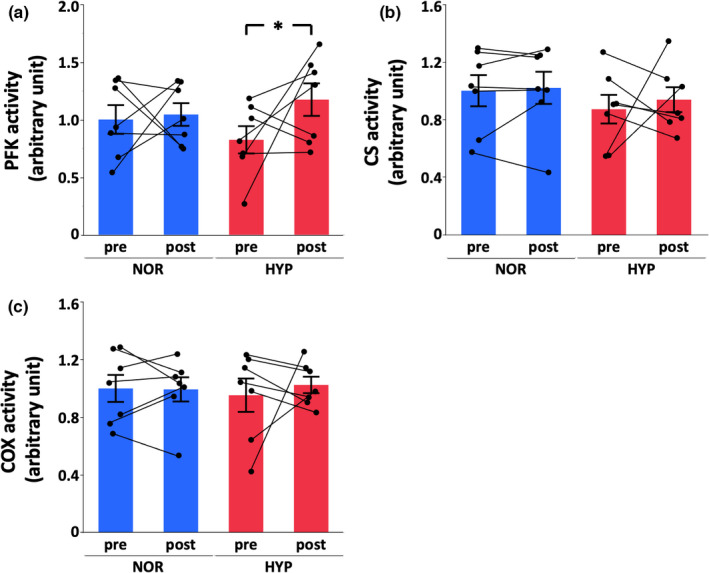
Phosphofructokinase (PFK; a), citrate synthase (CS; b), and cytochrome *c* oxidase (COX; c) activities at pre‐training and post‐training in normoxia (NOR) or hypoxia (HYP). Values are mean ± SE. *Significant changes from pre‐training (*p* < 0.05)

Whereas MCT1 protein expression did not change in both groups (NOR, −2%, *p* = 0.86, statistical power = 0.053; HYP, +9%, *p* = 0.44, statistical power = 0.10) after 4 weeks of training, MCT4 protein increased only in HYP (NOR, +4%, *p* = 0.22, statistical power = 0.19; HYP, +13%, *p* = 0.034, statistical power = 0.53) (Figure 4). Similar to MCT4 protein expression, PFK activity increased only in HYP after 4 weeks of training (NOR, +4%, *p* = 0.82, statistical power = 0.054; HYP, +42%, *p* = 0.045, statistical power = 0.28) (Figure 5). In contrast to glycolytic enzyme activity, CS (NOR, +2%, *p* = 0.87, statistical power = 0.063; HYP, +8%, *p* = 0.57, statistical power = 0.065) and COX (NOR, +1%, *p* = 0.95, statistical power = 0.051; HYP, +9%, *p* = 0.54, statistical power = 0.070) activities did not change in both groups after training (Figure 5).

## 4 DISCUSSION

The main findings of this study are that moderate‐intensity training in hypoxia for 4 weeks increased run time and $\dot{V}O_{2\max}$ at IET, MCT4 protein expression, and PFK activity in skeletal muscle, whereas the same training in normoxia only increased $\dot{V}O_{2\max}$. However, most cardiovascular and oxidative parameters including ${\dot{Q}}_{\text{max}}$, *SV*_max_, *C*_a‐v_O_2_, [*Hb*] at IET, MCT1 protein content, and CS and COX activities in skeletal muscle did not change significantly in both groups. Additional effects of hypoxic training at moderate‐intensity appear to be limited on exercise performance and glycolytic capacity in skeletal muscle, which yields a potential option for racehorse training strategy.

### 4.1 Effects on exercise performance and aerobic capacity after hypoxic training

Our previous studies reported that 4 weeks of high‐intensity training (100% $\dot{V}O_{2\max}$ 2 min, 3 sessions/week) in moderate hypoxia (15% O_2_) and similar hypoxic training (95% $\dot{V}O_{2\max}$ 2 min, 3 sessions/week, 4 weeks) in moderate hypoxia (16% O_2_) increased run time at IET by +28% and +21%, respectively (Mukai et al., 2020, 2021). Consistent with the previous reports in horses, we also observed a significant increase in run time only in HYP (+10%), but not in NOR. These results indicate that hypoxic training has an additional effect on exercise performance even at moderate‐intensity in horses. However, the increase in $\dot{V}O_{2\max}$ was similar in HYP (+4.3%) and NOR (+6.4%) and the changes in cardiovascular variables including ${\dot{Q}}_{\text{max}}$, *SV*_max,_ and *C*_a‐v_O_2_ were not significant and similar in both groups after training. Consistent with previous studies on LLTH training (Mukai et al., 2020, 2021), we did not observe any increase in [*Hb*], and the hypoxic exposure duration (<5 min) in this study appears to be too short to induce hematological changes. These results suggest that the greater improvement in performance in HYP may not be attributed to the changes in aerobic, cardiovascular, or hematological adaptations between HYP and NOR. At the same absolute exercise intensity and duration, exercise in hypoxia limits aerobic energy production and increases glycolytic energy contribution (Ohmura et al., 2020). The reduced O_2_ availability during hypoxic exercise may stimulate glycolytic capacity and metabolism, and lead to improve exercise performance. Furthermore, the relative exercise intensity in hypoxia is higher compared to the same absolute exercise intensity in normoxia, which may induce greater physiological adaptations after hypoxic training.

There was no significant difference in run time between NOR and HYP and the increase in run time in HYP was relatively smaller in this study compared to our previous results of high‐intensity hypoxic training despite the same/similar *F*_I_O_2_ and the same training duration and frequency being used (Mukai et al., 2020, 2021). The smaller improvements in performance may be due to the lower training intensity compared to our previous studies, as McLean et al. (McLean et al., 2014) stated that improvements in exercise performance at sea level appear most likely after high‐intensity and short‐term training in hypoxia.

Our previous study reported that 4 weeks of high‐intensity training (95% $\dot{V}O_{2\max}$ for 2 min, 3 days/week) in hypoxia (*F*_I_O_2_ = 16%) induces greater weight loss compared to the same training in normoxia (hypoxic group, −4.7%; normoxic group, −2.4%) (Mukai et al., 2021). However, in this study, we observed smaller weight loss in both groups (NOR, −1.3%; HYP, −1.7%) and no differences between groups. Therefore, we assume that the effect of moderate‐intensity training in hypoxia on body weight may be smaller compared to high‐intensity hypoxic training.

### 4.2 MCT proteins and glycolytic/mitochondrial enzyme activities after hypoxic training

We set the training intensity at 70% $\dot{V}O_{2\max}$, which is regarded as moderate intensity, but the training intensity in this study was determined at IET in normoxia, and not in hypoxia. As a result, arterial O_2_ saturation in HYP was reduced to 75% during exercise even at moderate intensity (Table 1), and a lower rate of O_2_ delivery to skeletal muscle increases metabolic stress on glycolytic pathway, which may stimulate the upregulation of glycolytic capacity and enzyme activity. Hypoxia‐inducible factor (HIF)‐1α has been reported to regulate glycolytic enzyme activity and MCT4 protein expression in skeletal muscle (Abe et al., 2015), which suggests that hypoxic training may improve energy production efficiently via glycolytic pathway. Puype et al. (2013) has reported that 6 weeks of sprint interval training in hypoxia (*F*_I_O_2_ = 14.4%) in humans increased muscle PFK activity more than normoxic training. Previously, we demonstrated that 2 weeks of high‐intensity training in mild hypoxia (*F*_I_O_2_ = 18%) increased MCT4 protein content and PFK activity (Wang et al., 2020) in horses, and the results in this study also indicate that similar training effects can occur even at moderate training intensity (70% $\dot{V}O_{2\max}$). In normoxia, 18 weeks of high‐intensity training (90–110% $\dot{V}O_{2\max}$ for 3 min, 5 sessions/week) is needed to increase MCT4 protein content, and the following 6 weeks of moderate‐intensity training (70% $\dot{V}O_{2\max}$ for 3 min, 5 sessions/week) cannot maintain the elevated MCT4 protein expression (Kitaoka et al., 2011). We also reported that PFK activity increased only after 12 weeks of high‐intensity training (80–100% $\dot{V}O_{2\max}$ for 5 min × 2, 5 sessions/week) in normoxia, not after 4 weeks of training (Eto et al., 2004). Collectively, long‐term high‐intensity training appears to be essential to enhance glycolytic capacity in normoxia, whereas even short‐term moderate‐intensity training can improve glycolytic energy metabolism if combined with hypoxic conditions. On the other hand, there was no significant difference in MCT4 between NOR and HYP groups after training. There are large individual variations in adaptation to hypoxic training in horses as we have previously reported (Mukai et al., 2020). Although further studies are needed to investigate the mechanism of MCT4 expression in hypoxic training, hypoxic training at moderate‐intensity can be a potential option for racehorse training strategy.

In contrast, MCT1 protein content and mitochondrial oxidative enzyme activities were unchanged in both NOR and HYP in the present study. A previous study in rats reported that MCT1 did not alter over 3 weeks of training at moderate running speed, whereas MCT1 in the soleus and red gastrocnemius muscles increased with more intense training for 3 weeks (Baker et al., 1998). In addition, our previous study reported no changes on MCT1 protein expression and COX activity after 2 weeks of high‐intensity hypoxic training in horses (Wang et al., 2020). These previous findings are consistent with the present results on MCT1, CS, and COX activities, which indicate that moderate‐intensity hypoxic training does not enhance MCT1 protein expression and oxidative energy metabolism in skeletal muscle.

### 4.3 Lactate threshold and MCT after hypoxic training

Contrary to our expectation, the lactate threshold (*V*_LA4_) in HYP did not increase despite *V*_LA4_ in NOR and MCT4 protein content in HYP increased after 4 weeks of training. These results are not consistent with previous human studies that reported greater or similar improvements in lactate threshold at IET after hypoxic training compared to normoxic training (Brechbuhl et al., 2018; Morton & Cable, 2005) and found no increase in muscle MCT4 protein after hypoxic training (Millet et al., 2014). In horses, an improvement in the lactate threshold was not always observed after hypoxic training, even if exercise performance in the hypoxic group showed a greater improvement than that of the nomoxic group (Mukai et al., 2020). Horses have a high ratio of type II fibers, in which lactate is mainly produced and MCT4 is localized, and, therefore, MCT4 may play a major role via lactate shuttle during exercise in horses. These differences in skeletal muscle properties and lactate transporter activity may induce different physiological adaptations after hypoxic training between horses and humans. Theoretically, MCT4 transports lactate from muscle fibers into blood stream and/or adjacent muscle fibers during exercise, so that the increase in MCT4 protein content can lead to an increase in plasma lactate concentration during submaximal exercise at IET, which would result in worsening *V*_LA4_. Furthermore, previous studies reported that positive correlations between *V*_LA4_ and $\dot{V}O_{2\max}$ (r = 0.79) (Eaton et al., 1999) and between CS activity and MCT1 protein content (r = 0.77) (Kitaoka et al., 2011), but not MCT4. These results indicate that *V*_LA4_ and MCT1 are closely related with aerobic capacity and MCT4 is associated with glycolytic capacity rather than aerobic capacity.

## 5 CONCLUSIONS

In summary, 4 weeks of moderate‐intensity hypoxic training improved exercise performance and glycolytic capacity in skeletal muscle in horses. While previous studies in horses have suggested that long‐term high‐intensity training is needed to increase MCT4 protein expression and glycolytic enzyme activities in normoxia, hypoxic training can induce these adaptations even in short‐term and at moderate‐intensity, which would provide more efficient training options to racehorse trainers compared with conventional training programs in normoxia.

## CONFLICT OF INTEREST

KM, HO, YT, and TT are employees of the Japan Racing Association.

## AUTHOR CONTRIBUTION

Conceptualization: KM, YK, and TT. Investigation: KM, YK, YT, TT, KT, and HO. Methodology: KM, YK, HO, KT, and TT. Writing ‐ original draft: KM. Writing ‐ review & editing: KM, YK, and HO.
